# Supplementary material for: Effect of the Consumption of Alcohol-Free Beers with Different Carbohydrate Composition on Postprandial Metabolic Response
Source: Nutrients. 2022 Feb 28;14(5):1046. doi: 10.3390/nu14051046 (PMC8912682; doi:10.3390/nu14051046)
Supplement: Supplementary file 1 [file nutrients-14-01046-s001.zip › Supplemental Tables and Figures.pdf]

Table S1. Nutritional composition of alcohol-free beers used in the study. .

	Alcohol-free beer with almost eliminated maltose and enriched with isomaltulose and maltodextrin (IMB)	Alcohol-free beer with almost eliminated maltose and enriched with maltodextrin (MB)	Alcohol-free beer with regular composition (RB)
Extract, % p/p	5.27	5.20	5.95
Total carbohydrates, % p/p	4.12	3.20	5.59
Sugars, % p/p	2.77	0.31	2.07
Glucose, % p/p	0.07	0.13	0.48
Fructose, % p/p	0.03	0.05	0.19
Sucrose, % p/p	<0.01	<0.01	0.05
Lactose, % p/p	<0.01	<0.01	1.00
Maltose, % p/p	0.07	0.13	1.35
Isomaltulose, % p/p	2.60	<0.01	<0.01
Glicerine, g/l	0.60	1.10	0.20
Ash, p/p	0.05	0.09	0.11
Sodium, mg/kg	34	57.00	41
Citric acid, mg/kg	73	133.00	100
Density, g/ml	1.02	1.02	1.02

Table S2. Variation of clinical and anthropometric characteristics of subjects included in study 1.[1.]

	Glucose (25g of CH)	RB (25g of CH)	MB (25g of CH)	IMB (25g of CH)	p^2
Body weight, kg	74.0 (62.3- 77.7)	74.3 (59.8 – 77.0)	75.2 (64.4 – 78.0)	76.3 (72.8 – 77.4)	0.934
Systolic blood pressure, mmHg	119 (104 – 121)	106 (104 – 112)	114 (101 – 123)	117 (104 – 123)	0.897
Diastolic blood pressure, mmHg	77.0 (68.5 – 85.0)	72.0 (68.0- 77.0)	75.0 (63.0 – 79.0)	74.0 (67.0- 78.0)	0.694
Consumption time of each drink (min)	6.00 (5.00 – 8.00)	7.00 (6.00 – 12.0)	18.0 (15.3 – 22.0)	13.0 (9.00 – 15.0)	0.012
Physical activity					
Vigorous physical activity, METs/min	0.00 (0.00 – 600)	1140 (180- 1920)	1280 (720 – 1890)	120 (0.00 – 1680)	0.722
Moderate physical activity, METs/min	0.00 (0.00 – 135)	0.00 (0.00- 800)	0.00 (0.00- 360)	0.00 (0.00 – 240)	0.811
Walking, METs/min	1386 (1213 – 2772)	1732 (1386 – 2599)	1386 (1040- 2079)	1213 (322 – 1534)	0.565
Global physical activity, METs/min	2969 (1568 - 4073	3533 (2346 – 5119)	3335 (1546 – 4599)	2431 (1170 – 4566)	0.806

[1] Variables are expressed as median (25th percentile - 75th percentile). CH stands for carbohydrates; IMB: alcohol-free beer with almost completely fermentation of regular CH and enriched with isomaltulose (2.5 g/100 mL) and a resistant maltodextrin (0.8 g/100 mL); MB: alcohol-free beer with almost completely fermentation of regular CH and enriched with maltodextrin (2.0 g/100 mL); RB: regular alcohol-free beer.[2] The *p*-value was calculated by Kruskal-Wallis test.

Table S3. Variation of clinical and anthropometric characteristics of subjects included in study 2.[1.]

	RB + 50g of CH from white bread (64.3g of CH)	IMB + 50g of CH from white bread (64.3g of CH)	MB + 50g of CH from white bread (64.3g of CH)	Water + 50g of CH from white bread (50g of CH)	Water + 64.3 g of CH from white bread (64.3g of CH)	*p*
Body weight, kg	78.7 ± 17.1	77.3 ± 17.0	78.0 ± 17.0	77.7 ± 16.7	73.7 ± 20.9	0.279
Systolic blood pressure, mmHg	114 ± 10.8	105 ± 27.9	112 ± 12.9	119 ± 11.5	116 ± 11.2	0.108
Diastolic blood pressure, mmHg	73.6 ± 8.01	69.9 ± 18.7	71.4 ± 7.83	75.9 ± 6.57	73.0 ± 7.46	0.866
Consumption Time of each drink (min)	11.8 ± 3.78	11.0 ± 4.01	12.3 ± 5.32	9.95 ± 4.37	11.6 ± 3.84	0.379
Physical activity						

Vigorous physical activity, METs/min	1138 ± 1480	1488 ± 1574	1032 ± 2010	1726 ± 1839	852 ± 1313	0.358
Moderate physical activity, METs/min	554 ± 867	548 ± 1247	586 ± 1146	653 ± 1606	556 ± 875	0.974
Walking, METs/min	2453 ± 2475	2512 ± 2265	1701 ± 1777	1837 ± 1688	1970 ± 1943	0.605
Global physical activity, METs/min	4145 ± 2699	4548 ± 3493	3319 ± 2413	4216 ± 2550	3378.1 ± 2515	0.508

[1]Variables are expressed as mean ± standard deviation. CH stands for carbohydrates; IMB: alcohol-free beer with almost completely fermentation of regular CH and enriched with isomaltulose (2.5 g/100 mL) and a resistant maltodextrin (0.8 g/100 mL); MB: alcohol-free beer with almost completely fermentation of regular CH and enriched with maltodextrin (2.0 g/100 mL); RB: regular alcohol-free beer. [2]The *p*-value was calculated by ANOVA test.

Table S4. Dietary characteristics of the three days prior to the consumption of each type of beer in the study 1.

	Glucose (25g of CH)	RB (25g of CH)	MB (25g of CH)	IMB (25g of CH)	*p*
Energy, kcal	2184 ± 758	2301 ± 956	2462 ± 870	2195 ± 447	0.924
Carbohydrates, %	181 ± 76.5	207 ± 103	218 ± 109	189 ± 61.2	0.864
Sugars, g	73.9 ± 30.4	74.7 ± 32.2	73.1 ± 33.3	84.4 ± 15.5	0.891
Polysaccharides, g	105 ± 51.4	128 ± 74.1	143 ± 79.3	101 ± 51.5	0.625
Fiber, g	19.0 ± 9.37	21.5 ± 13.9	24.5 ± 16.4	20.3 ± 6.35	0.854
Fat, %	109 ± 42.6	107 ± 32.5	120 ± 31.9	110 ± 31.9	0.928
Saturated fatty acids, %	35.9 ± 15.8	33.3 ± 9.32	32.3 ± 9.94	32.0 ± 7.20	0.903
Monounsaturated fatty acids, %	47.1 ± 20.1	44.8 ± 16.9	52.1 ± 13.0	45.9 ± 16.1	0.895
Polyunsaturated fatty acids, %	15.9 ± 8.33	18.4 ± 9.98	23.5 ± 13.5	21.2 ± 9.57	0.527
Cholesterol, mg	507 ± 252	374 ± 151	445 ± 185	349 ± 136	0.363
Protein, %	110 ± 45.7	102 ± 42.9	122 ± 43.3	107 ± 31.8	0.862
Sodium, mg	3208 ± 1182	3325 ± 1358	4383 ± 2120	3118 ± 1082	0.422

[1]Variables are expressed as mean ± standard deviation. CH stands for carbohydrates; IMB: alcohol-free beer with almost completely fermentation of regular CH and enriched with isomaltulose (2.5 g/100 mL) and a resistant maltodextrin (0.8 g/100 mL); MB: alcohol-free beer with almost completely fermentation of regular CH and enriched with maltodextrin (2.0 g/100 mL); RB: regular alcohol-free beer. [2]The *p*-value was calculated by ANOVA test.

Table S5. Dietary characteristics of the three days prior to the consumption of each type of the intervention in the study 2.

	White bread reference (50g HC)	White bread + RB (64,3 g HC)	White bread + MB (64,3 g HC)	White bread + IMB (64,3g HC)	White bread + plus white bread (64,3g HC)	*p*
Energy, kcal	2057 ± 655	2035 ± 758	2025 ± 626	1963 ± 689	2029 ± 601	0.994
Carbohydrates, %	178 ± 69.8	188 ±78.7	190 ± 77.5	182 ± 73.3	182 ± 69.5	0.985
Sugars, g	71.2 ± 26.0	68.2 ±25.4	76.9 ±32.3	77.5 ± 26.5	73.8 ± 31.6	0.825
Fiber, g	16.8 ± 9.19	17.0 ± 6.57	16.9 ± 8.13	19.0 ± 8.60	107 ± 51.7	0.807
Fat, %	106 ± 38.2	95.2 ± 38.1	95.7 ± 29.3	87.7 ± 35.5	100 ± 34.7	0.593
Saturated fatty acids, %	33.8 ± 15.6	30.6 ± 14.4	30.8 ± 11.7	26.5 ± 13.2	31.2 ±12.6	0.574
Monounsaturated fatty acids, %	44.3 ± 16.2	38.5 ± 14.6	39.8 ±10.6	39.0 ±15.4	42.4 ± 16.5	0.709
Polyunsaturated fatty acids, %	18.1 ± 8.40	17.6 ± 9.84	14.7 ± 5.31	14.3 ± 7.38	17.5 ± 6.18	0.368
Cholesterol, mg	378 ± 160	409 ± 235	389 ± 243	408 ± 220	358 ± 199	0.940

Protein, %	94.8 ± 30.4	93.3 ± 32.6	87.9 ± 26.6	101 ± 32.5	93.0 ± 26.5	0.744
Sodium, mg	2914 ± 1475	2973 ±971	2939 ± 1277	3037 ± 1601	2651 ± 1108	0.911

[1]Variables are expressed as mean ± standard deviation. CH stands for carbohydrates; IMB: alcohol-free beer with almost completely fermentation of regular CH and enriched with isomaltulose (2.5 g/100 mL) and a resistant maltodextrin (0.8 g/100 mL); MB: alcohol-free beer with almost completely fermentation of regular CH and enriched with maltodextrin (2.0 g/100 mL); RB: regular alcohol-free beer. [2]The *p*-value was calculated by ANOVA test.

Table S6. AUCs of glucose, insulin, GIP and GLP-1 produced after the consumption of each drink in the study 1.[1.]

**GLUCOSE**	**AUC**	**95% CI**	p^2	p^3
RB (25 g of CH)	11578 ± 313	12211 to 10944	Ref.	0.027
IMB (25g of CH)	11415 ± 241	11898 to 10932	0.608	0.085
MB (25g of CH)	10631 ± 220	11077 to 10186	**0.016**	0.772
Glucose (25g of CH)	10721 ± 218	11162 to 10280	**0.027**	Ref.
**INSULIN**	**AUC**	**95% CI**	p^2	p^3
RB (25 g of CH)	1196 ± 103.5	983 to 1408	Ref.	0.648
IMB (25g of CH)	928 ± 51.3	824 to 1032	**0.024**	**<0.001**
MB (25g of CH)	937.6 ± 58.6	816 to 1059	**0.036**	**<0.001**
Glucose (25g of CH)	1249 ± 50.7	1145 to 1353	0.648	Ref.
**GIP**	**AUC**	**95% CI**	p^2	p^3
RB (25 g of CH)	10065 ± 545	8961 to 11169	Ref.	0.077
IMB (25g of CH)	6518 ± 324	5865 to 7172	**<0.001**	**<0.001**
MB (25g of CH)	5795 ±250	5290 to 6300	**<0.001**	**<0.001**
Glucose (25g of CH)	11801 ± 793	10180 to 13423	0.077	Ref.
**GLP-1**	**AUC**	**95% CI**	p^2	p^3
RB (25 g of CH)	17402 ± 579	16246 to 18558	Ref.	<0.001
IMB (25g of CH)	16929 ± 639	15654 to 18203	0.584	**<0.001**
MB (25g of CH)	16633 ± 644	15341 to 17920	0.373	**<0.001**
Glucose (25g of CH)	13580 ± 421	12739 to 14422	<0.001	Ref.

[1]AUC stands for area under the curve; CH: carbohydrates; IMB: alcohol-free beer with almost completely fermentation of regular CH and enriched with isomaltulose (2.5 g/100 mL) and a resistant maltodextrin (0.8 g/100 mL); MB: alcohol-free beer with almost completely fermentation of regular CH and enriched with maltodextrin (2.0 g/100 mL); RB: regular alcohol-free beer. [2]The *p*-value denotes the comparison between the AUCs producing the two-to-two comparisons, using as reference the AUC produced after the consumption of 25 g of CH coming from RB and was calculated by the t-test in the PK package [3]The *p*-value denotes the comparison between the AUCs producing the two-to-two comparisons, using as reference the AUC produced after the consumption of 25 g of CH coming from glucose solution and was calculated by the t-test in the PK package.

Table S7. AUCs of glucose, insulin, GIP and GLP-1 produced after the consumption of each drink/mean in the study 2.[1.]

**GLUCOSE**	**AUC**	**95% CI**	p^2	p^3	p^4
RB + white bread (64.3g of CH)	14213 ± 179	13860 to 14568	0.055	0.204	Ref.
IMB + white bread (64.3g of CH)	13590 ± 139	13315 to 13865	0.451	0.159	**0.006**
MB + white bread (64.3g of CH)	13360 ± 133	13096 to 13624	0.067	**0.012**	**<0.001**

Water + white bread (64.3g of CH)	13901 ± 169	13568 to 14235	0.518	Ref.	0.055
Water+ white bread (50g of CH)	13751 ± 161	13433 to 14068	Ref.	0.518	0.204
**INSULIN**	**AUC**	**95% CI**	***p*[2]**	***p*[3]**	***p*[4]**
RB + white bread (64.3g of CH)	3115 ± 159	2798 to 3432	**<0.001**	**0.043**	Ref.
IMB + white bread (64.3g of CH)	2847 ± 149	2552 to 3142	**0.017**	0.128	0.229
MB + white bread (64.3g of CH)	2664 ± 127	2412 to 2915	**0.016**	0.554	**0.028**
Water + white bread (64.3g of CH)	2564 ± 105	2356 to 2771	**0.042**	Ref.	**0.043**
Water+ white bread (50g of CH)	2234 ± 122	1993 to 2476	Ref.	**0.042**	**<0.001**
**GIP**	**AUC**	**95% CI**	***p*[2]**	***p*[3]**	***p*[4]**
RB + white bread (64.3g of CH)	25774 ± 1085	23627 to 27922	**<0.001**	**0.019**	Ref.
IMB + white bread (64.3g of CH)	22545 ± 1194	20175 to 24915	**<0.001**	0.857	**0.046**
MB + white bread (64.3g of CH)	23189 ± 1116	20978 to 25399	**<0.001**	0.540	0.098
Water + white bread (64.3g of CH)	22263 ± 1015	20244 to 24282	**<0.001**	Ref.	**0.019**
Water+ white bread (50g of CH)	15994 ± 552	14902 to 17086	Ref.	**<0.001**	**<0.001**
**GLP-1**	**AUC**	**95% CI**	***p*[2]**	***p*[3]**	***p*[4]**
RB + white bread (64.3g of CH)	20813 ± 614	19599 to 22027	<0.001	0.074	Ref.
IMB + white bread (64.3g of CH)	18338 ± 542	17266 to 19410	**0.005**	0.119	**0.028**
MB + white bread (64.3g of CH)	19560 ± 585	18404 to 20715	**<0.001**	0.874	0.141
Water + white bread (64.3g of CH)	19443 ± 453	18547 to 20339	**<0.001**	Ref.	0.074
Water+ white bread (50g of CH)	16286 ± 489	15320 to 17253	Ref.	**<0.001**	**<0.001**

[1]AUC stands for area under the curve; CH: carbohydrates; IMB: alcohol-free beer with almost completely fermentation of regular CH and enriched with isomaltulose (2.5 g/100 mL) and a resistant maltodextrin (0.8 g/100 mL); MB: alcohol-free beer with almost completely fermentation of regular CH and enriched with maltodextrin (2.0 g/100 mL); RB: regular alcohol-free beer. [2]The *p*-value denotes the comparison between the AUCs producing the two-to-two comparisons, using as reference the AUC produced after the consumption of 50 g of CH and was calculated by the t-test in the PK package.[3]The *p*-value denotes the comparison between the AUCs producing the two-to-two comparisons, using as reference the AUC produced after the consumption of 64.3 g of CH, all of them coming from white bread and was calculated by the t-test in the PK package [4]The *p*-value denotes the comparison between the AUCs producing the two-to-two comparisons, using as reference the AUC produced after the consumption of 64.3 g of CH, coming from RB + 50 g of CH from white bread, and was calculated by the t-test in the PK package.

.

Supplemental Figure 1. Lipid metabolism parameters determined in the study 1

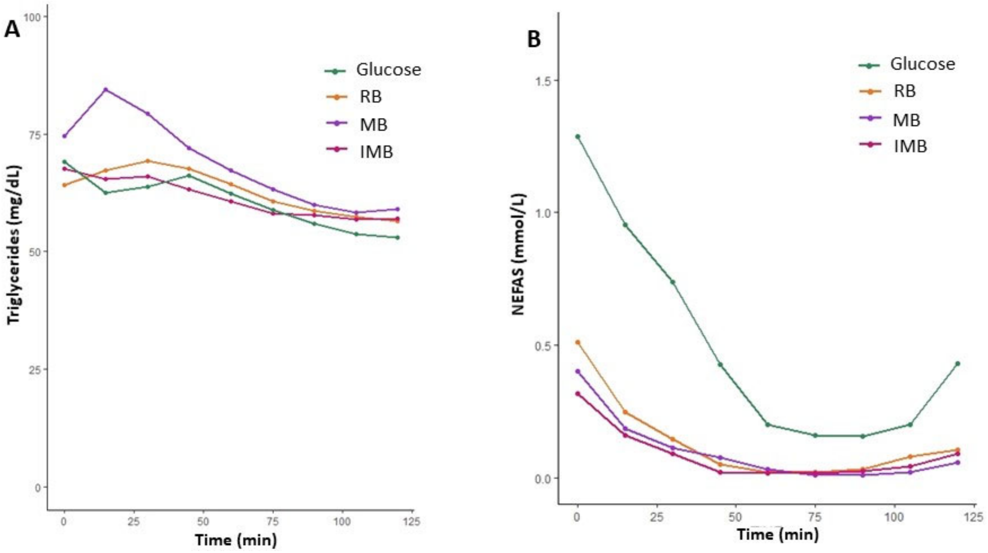

Figure S1. Lipid metabolism parameters determined in the study 1.

Supplemental Figure 2. Lipid metabolism parameters determined in the study 2

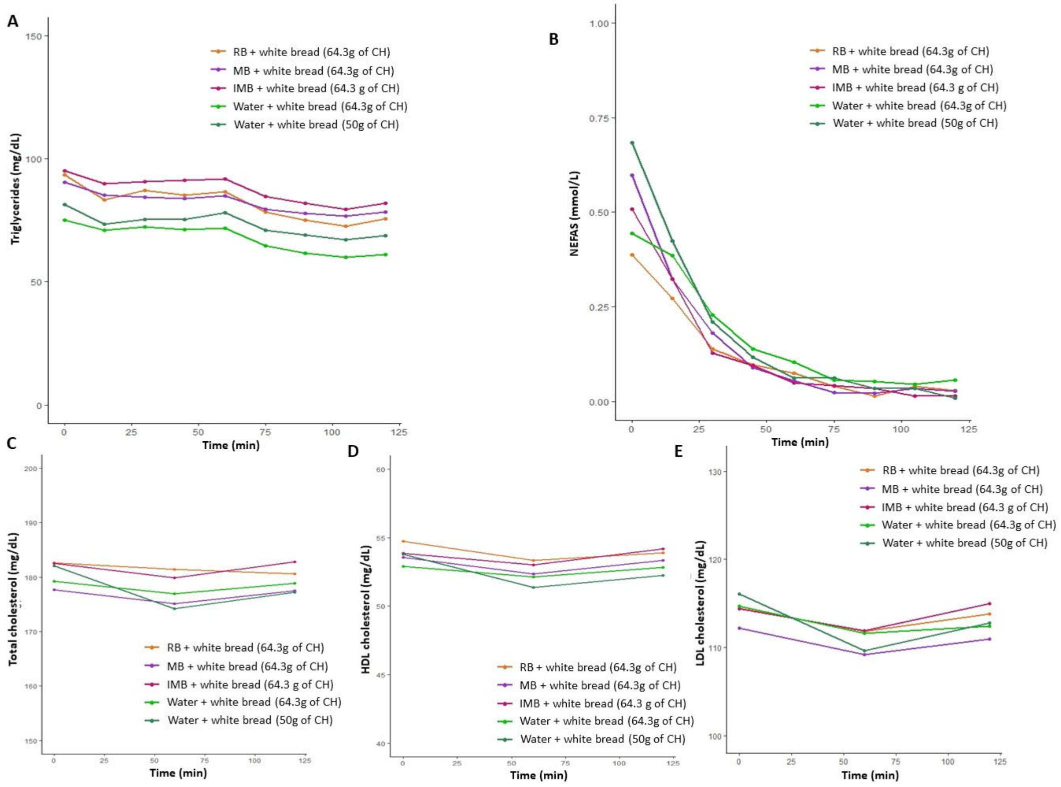

Figure S2. Lipid metabolism parameters determinded in the study 2.
